# The association of body mass index variability with cardiovascular disease and mortality: a mediation analysis of pooled cohorts

**DOI:** 10.3389/fendo.2024.1345781

**Published:** 2024-05-13

**Authors:** Ladan Mehran, Mohammadjavad Honarvar, Safdar Masoumi, Davood Khalili, Fereidoun Azizi, Michael J. Blaha, Atieh Amouzegar

**Affiliations:** ^1^ Endocrine Research Center, Research Institute for Endocrine Sciences, Shahid Beheshti University of Medical Sciences, Tehran, Iran; ^2^ Prevention of Metabolic Disorders Research Center, Research Institute for Endocrine Sciences, Shahid Beheshti University of Medical Sciences, Tehran, Iran; ^3^ Department of Biostatistics and Epidemiology, Research Institute for Endocrine Sciences, Shahid Beheshti University of Medical Sciences, Tehran, Iran; ^4^ Ciccarone Center for the Prevention of Cardiovascular Disease, Johns Hopkins University, Baltimore, MD, United States; ^5^ Department of Epidemiology, Johns Hopkins Bloomberg School of Public Health, Johns Hopkins University, Baltimore, MD, United States

**Keywords:** cardiovascular disease, mortality, body mass index, weight variability, mediation analysis

## Abstract

**Aim:**

We aimed to investigate the effect of BMI variability on CVD and mortality and to explore the mediation effects of the main cardiovascular risk factors contributing to this association.

**Method:**

Participants aged 40-65 years were pooled from three cohort studies(ARIC [Atherosclerosis Risk in Communities], MESA [Multi-ethnic Study of Atherosclerosis], and TLGS [Tehran Lipid and Glucose Study]. We employed root mean squared error of the fractional mixed model to calculate BMI variability in the measurement period. In the event assessment period, the hazard ratios for CVD and mortality were estimated using Cox proportional hazard regression models. In the next step, the mediation and interaction effects of fasting plasma glucose, total cholesterol, and systolic blood pressure were determined.

**Results:**

A total of 19073 participants were included in this pooled analysis. During a median of 20.7 years of follow-up, 3900 (20.44%) CVD and 6480 (33.97%) all-cause mortality events were recorded. After adjusting for potential confounders, BMI variability was linked to the 1.3 (1.2-1.4) and 1.7 (1.6-1.8) increased risk of CVD and mortality, respectively. Fasting plasma glucose mediated approximately 24% and 8% of the effect of BMI variability on CVD and mortality, respectively. However, systolic blood pressure and total cholesterol did not have mediation effects in this association.

**Conclusion:**

High BMI variability is independently associated with the development of CVD and mortality. This association is partly mediated through fasting plasma glucose. Modern cardiometabolic therapies that lower fasting glucose may reduce the risk of future CVD and mortality in individuals with high BMI variability.

## Introduction

Obesity is a well-known risk factor for cardiovascular disease (CVD) and mortality (1, 2). Overweight and obese individuals with additional cardiovascular risk factors are recommended to lose weight through lifestyle modifications (3). However, weight loss maintenance is challenging and is followed by weight regain in the long term (4). In a systematic review of observational studies, 42% of the general population reported personal weight control attempts (5). Adherence to a weight loss strategy plan, metabolic adaptation, energy homeostasis, and pregnancy are the factors that may lead to recurrent cycles of weight loss and regain and, thus, unsuccessful attempts at sustained weight loss (6).

The obesity paradox refers to the seemingly counterintuitive notion that body mass index (BMI) is not a consistent cardiovascular risk factor in overweight and obese individuals (7, 8), and questions the cardiovascular health benefits of weight loss in the long term (9–11). Body weight variability has been examined as an additional risk factor to explain the controversial findings on the effect of weight loss on CVD and mortality (12, 13). Although several studies suggested that weight variability independently increases the risk of CVD and mortality (12), some studies found no association or decreased risk for future CVD (14–16). In addition, the relationship between weight variability and CVD is inconsistent among different subpopulations (17, 18). The mechanisms through which weight variability increases the risk of CVD and mortality are not understood, and a few studies have explored the correlation of weight variability with cardiovascular risk factors (19, 20).

To address the gap in the literature, the current study investigated the association of BMI variability with CVD incidence and all-cause mortality in a large pooled sample and in different subpopulations. We also delved deeper into the underlying mechanisms driving the link between BMI variability and CVD and mortality by performing mediation analyses. This would help to determine the specific cardiovascular risk factors that act as intermediaries in this relationship, shedding new light on the potentially complex relationship between BMI variability and CVD and mortality.

## Methods

### Study population

The current study was based on data from three large population-based cohort studies designed to investigate the risk factors for non-communicable diseases: Atherosclerosis Risk in Communities (ARIC), Multi-Ethnic Study of Atherosclerosis (MESA), and Tehran Lipid and Glucose Study (TLGS). The Biologic Specimen and Data Repository Information Coordinating Center (BioLINCC), managed by the National Heart, Lung, and Blood Institute (NHLBI), granted access to the public-use datasets for the ARIC and MESA studies by coordinating their storage and distribution. Previous publications have provided detailed and comprehensive descriptions of the cohorts’ design, protocols, and participant characteristics (21–23).

### ARIC

The ARIC study is a prospective multi-center cohort of 15,972 adults aged 45-64 in 1987-1989, randomly selected and recruited from four clinical sites in the United States (Washington County, MD; Forsyth County, NC; Jackson, MS; and Minneapolis, MN). The study participants were enrolled in seven study examination visits with three-year intervals (visit 1: 1987-1989, visit 2: 1990–1992, visit 3: 1993–1995, visit 4: 1996–1998, visit 5: 2011–2013, visit 6: 2016–2017, and visit 7: 2018–2019) and followed annually through telephone interviews to obtain most recent health-related information.

### MESA

In 2000-2002, the MESA study recruited a population-based sample of 6,814 individuals between 45 and 84 years old from six field centers across the United States (Baltimore, MD; Chicago, IL; Forsyth County, NC; Los Angeles, CA; New York, NY; and Saint Paul, MN). The subsequent follow-up examination visits were performed during 2002-2004 (visit 2), 2004-2005 (visit 3), 2005-2007 (visit 4), and 2010-2012 (visit 5) and 2016-2018 (visit 6). The participants were contacted at 9-12 months intervals for updates on medical conditions.

### TLGS

The TLGS is an ongoing population-based cohort study initiated during 1999-2001 by recruiting 18,555 members aged≥3 years in Tehran, Iran. The examination visits were held in approximate three-year intervals (visit 2: 2002-2005, visit 3: 2005-2008, visit 4: 2009-2011, visit 5: 2011-2014, visit 6: 2015-2018). Participants were followed annually for any medical event by telephone calls.

### Study timeline

**Figure 1:**
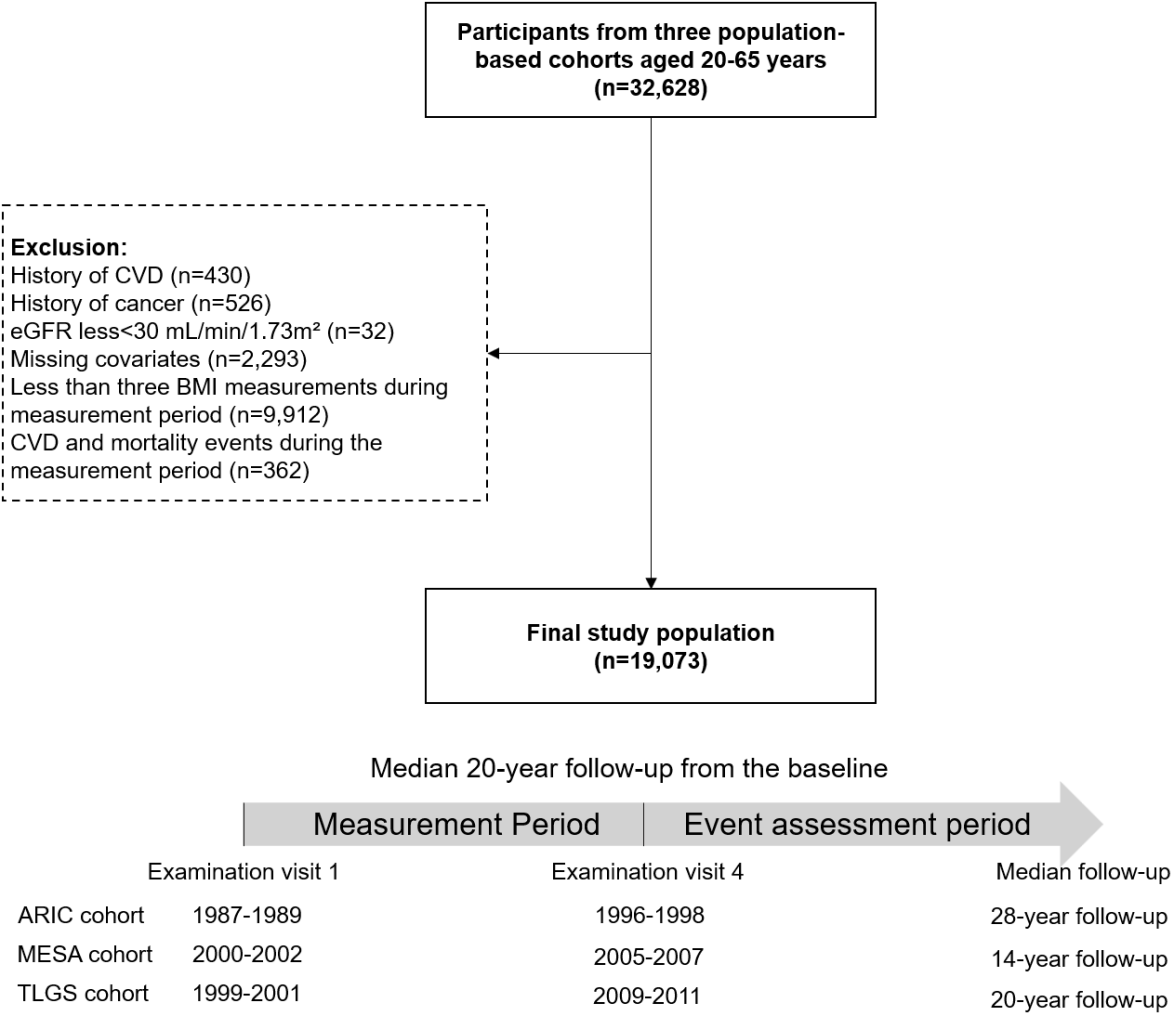
Flowchart of the study participants and timeline of the pooled study cohort. CVD, cardiovascular disease; eGFR, estimated glomerular filtration rate; BMI, body mass index; ARIC, Atherosclerosis Risk in Communities; MESA, Multi-Ethnic Study of Atherosclerosis; TLGS, Tehran Lipid and Glucose Study.

The timeline for the current study was divided into two distinct periods: a measurement period, beginning at baseline and continuing until the end of the fourth examination visit, and an event assessment period, which started from the fourth examination until the end of the study (**Figure 1**). We included a total of 32628 participants aged 40 to 65 years old at baseline from the ARIC (n=14996), MESA (n=4084), and TLGS (n=13548) cohorts. Participants with CVD (n=430), cancer (n=526), estimated glomerular filtration rate (eGFR)<30 mL/min/1.73m² (n=32), and missing covariates at baseline (n=2293) were excluded. Participants with CVD and mortality events in the measurement period (n=362) and with less than three BMI records during the measurement period (n=9912) were also excluded.

### Definition of variables

BMI was calculated by dividing weight in kilograms by the square of height in meters (kg/m²). Diabetes was defined as fasting plasma glucose (FPG) 126 mg/dl or the use of glucose-lowering medication. Hypertension was determined by systolic blood pressure (SBP)≥140mmHg, diastolic blood pressure≥90mmHg, or the use of antihypertensive medications. The education level categories were: 1) primary school (less than 6 years of education), 2) high school (6-12 years of education), and 3) higher education degree (12 years or more of education). Two categories were used to classify smoking status: current and non-smokers (former and never smokers). The eGFR was calculated using the Chronic Kidney Disease Epidemiology Collaboration (CKD-EPI) 2021 formula.

### Definition of outcomes

Our primary outcomes were incident CVD and all-cause mortality. Incident CVD was defined as a composite of fatal and non-fatal coronary heart disease (CHD) and stroke; incident CHD was defined as myocardial infarction, angina if followed by revascularization or medical treatment, coronary procedures, and death due to CHD. The outcome review committees in each study adjudicated the classification and incidence date of events to examine hospitalization and mortality based on previously published study protocols (21–23).

### Statistical analysis

The baseline characteristics of the study population were reported as mean values with standard deviation (SD) for continuous variables and as percentages for categorical variables. The data were compared using appropriate statistical tests, the Chi-square test for categorical variables and one-way analysis of variance (ANOVA) for continuous variables.

Our investigation in this study was based on the variability of BMI values instead of weight, as weight changes can be affected by variations in height. In the current study, we ran mixed effect regression models using fractional polynomials to obtain the BMI trend of each individual in the measurement period and calculated BMI variability using the root mean squared error (RMSE) (1). In this method, BMI variability is not sensitive to the mean values and number of measurements. Moreover, it captures large weight variations and not the non-linearity in the natural trend of BMI (18, 24).

### Estimation of BMI trend in longitudinal age

Briefly, we used mixed-effect regression models to investigate the BMI trend of each individual in longitudinal age since mixed-effect regression models account for the correlations that arise from the multiple BMI measurements taken from one person. This model estimates both the population-level effect (fixed effect; with age as the covariate) and the individual-level effect (random effect). By including the random effect, we also captured the variability of BMI change between individuals. We used fractional polynomials to obtain the best-fit mixed-effect regression model since the fractional polynomials is a flexible approach that allows the power of the polynomial terms to be any real number and fit curves that are not possible with traditional polynomials.

To estimate BMI trends, we fitted smooth trends using fractional polynomial mixed models (with random intercept) that accounted for the longitudinal age at each measurement. We derived the trend parameters for longitudinal age and intercept using random effects (Equation 1).


(1) $${\text{BMI}}_{i,j}=\left(\beta_{0}+b_{0i}\right)+\beta_{1}age_{ij}+\beta_{2}age_{ij}^{\;2}+\beta_{3}age_{ij}^{\;3}+{\text{ε}}_{ij}$$

In the equation, ${\text{BMI}}_{i,j}$ is the BMI of the participant “i” at the examination visit “j”. *β*_0_ represents the intercept and $b_{0i}$ represents the random intercept for each individual i with the assumption of normal distribution. This random intercept term accounts for individual variation that cannot be explained by the other variables in the model. The population parameters, $\beta_{1}-\beta_{3}$ , represent the estimated changes in BMI values over longitudinal age. The optimal model for the BMI trend of individuals was selected based on the fractional model. Equation (2) represents the final optimal model for predicting BMI values:


(2) $${\hat{\text{BMI}}}_{i,j}=\left({\hat{\beta }}_{0}+{\hat{b}}_{0i}\right)+{\hat{\beta }}_{1}age+{\hat{\beta }}_{2}age^{2}+{\hat{\beta }}_{3}age^{3}\;$$

### BMI variability calculation

After obtaining the BMI trend for each individual (the final model), we calculated the intraindividual BMI variability using the root mean squared error (RMSE) formula (Equation 3) i.e., the average of the square root of the difference between the actual and fitted BMI (obtained from the final model) values at each time point.


(3) $$RMSE_{i}=\sqrt{\frac{\sum_{j=1}^{k}{\left(BMI_{i,j}-{\hat{BMI}}_{i,j}\right)}^{2}}{N}\;\;}$$

BMI_i,j_ = Actual (observed) longitudinal BMI.

${\hat{\text{BMI}}}_{\text{i},\text{j}}$ = Estimated (fitted) longitudinal BMI.

i represents each individual in the dataset.

k represents the number of BMI records for an individual.

j represents the records of BMI measurement for each individual.

N represents the number of measurements for an individual.

### Survival analysis

To assess the relationship between BMI variability (expressed as BMI-RMSE) and the risk of study outcomes, multivariate-adjusted Cox proportional hazards regression models were utilized. Hazard ratios (HRs) and corresponding 95% confidence intervals (CIs) were calculated using the lowest tertile of BMI variability as the reference group. Model 1 was adjusted for age at baseline and sex. Model 2 was adjusted for age, sex, education level, premature CVD family history, and smoking status. Model 3 was adjusted for model 2 and baseline BMI, fasting plasma glucose, systolic blood pressure, and total cholesterol.

### Mediation analysis

We investigated the role of metabolic indices of FPG, total cholesterol (TC), and SBP, using their mean values in the measurement period, in explaining the effect of BMI variability on CVD incidence and all-cause mortality. To conduct mediation analysis, we performed preliminary analysis to evaluate the association of BMI variability with the potential mediators and the association of the potential mediators with the outcomes. We then conducted the mediation analysis using a four-way decomposition (25). In this method, the total effect of the exposure on the outcome can be divided into four components. The effect was attributed to both mediation and interaction, interaction only, mediation only, and neither mediation nor interaction. The lowest BMI variability tertile was considered the reference group, and the direct and indirect effect of BMI variability on CVD and mortality was calculated accordingly. The analysis was conducted using R-3.0.3 (R Foundation for Statistical Computing) and Med4way package in STATA 14.0 (StataCorp, College Station, TX, USA) (25).

## Results

**Table 1** presents the baseline characteristics of the study participants according to the BMI variability tertiles. This pooled cohort comprised 19073 participants (51.89% women) with a mean age of 53 ± 6 years at baseline. The value of BMI variability ranged from 0.02-0.63 Kg/m^2^ in the first, 0.63-1.07 Kg/m^2^ in the second, and 1.07-9.32 Kg/m^2^ in the third tertiles. Toward the highest tertile, the mean values of baseline BMI, SBP, FPG, TG, TC, and BMI change increased while the mean age decreased. The highest BMI variability tertile had a higher prevalence of women, individuals with obesity, and lower education levels, while the lowest BMI variability tertile showed a higher prevalence of current smokers.

**Table 1:** Baseline characteristics of participants of the pooled cohort.

Characteristic	BMI variability RMSE Tertiles					P-value
	Total	Tertile 1 (.0258, 0.638)	Tertile 2 (0.638, 1.076)		Tertile 3 (1.076, 9.32)	
**Number of participants**	19,073	6,359	6,362		6,352	
**Age (years)**	53 ± 6	53 ± 6	53 ± 6		53 ± 6	<0.001
**Female**	9897 (51.9)	3083 (48.5)	3229 (50.7)		3585 (56.4)	<0.001
**Premature CVD Family history**	1580 (8.3)	531 (8.3)	561 (8.82)		488 (7.7)	
Education level						<0.001
Illiterate/primary	5199 (27.3)	1516 (23.9)	1762 (27.7)		1921 (30.3)	
High school	8095 (42.5)	2661 (41.9)	2756 (43.4)		2678 (42.2)	
Higher education	5753 (30.2)	2173 (34.2)	1837 (28.9)		1743 (27.5)	
Current smoker						<0.001
Yes	1828 (12.5)	631 (13.3)	645 (13.4)		552 (11.0)	
No	12745 (87.5)	4120 (86.7)	4153 (86.6)		4472 (89.0)	
**BMI (Kg/m^2^)**	27.8 ± 5.2	26.30 ± 3.4	27.2 ± 4.7		30.1 ± 6.2	<0.001
BMI categories						<0.001
BMI<30 Kg/m^2^	13624 (71.4)	5424 (85.3)	4744 (74.8)		3456 (54.4)	
BMI≥30 Kg/m^2^	5449 (28.6)	935 (14.7)	1618 (25.4)		2896 (45.6)	
**SBP (mm Hg)**	120.7 ± 18.2	119.5 ± 17.6	120.6 ± 18.24		122.0 ± 18.7	<0.001
**DBP (mm Hg)**	72.4 ± 10.1	72.9 ± 10.0	72.1 ± 10.17		72.0 ± 10.2	<0.001
**FPG (mg/dl)**	103.9 ± 34.8	99.8 ± 26.4	102.7 ± 30.84		109.4 ± 43.97	<0.001
**TG (mg/dl)**	141.9 ± 99.8	137.2 ± 87.6	140.8 ± 95.3		147.9 ± 114.51	<0.001
**TC (mg/dl)**	212.3 ± 42.0	210.1 ± 40.1	212.7 ± 41.2		214.0 ± 44.6	<0.001

The categorical and continuous variables were reported as count (percentage) and mean ± SD, respectively.

BMI, body mass index; RMSE, root mean squared error; CVD, cardiovascular disease; SBP, systolic blood pressure; DBP, diastolic blood pressure; FPG, fasting plasma glucose; TG, triglyceride; TC, total cholesterol.

During a median of 20.7 years of follow-up, 3900 (20.45%) incident CVD events and 6480 (33.97%) deaths were recorded. **Table 2** displays the association of BMI variability with CVD events and mortality. After adjusting for age, sex, education, smoking status, and family history of CVD, the highest BMI variability tertile had increased risk for future CVD (HR:1.3; 95% CI:1.2 - 1.4) and mortality (HR: 1.7, 95% CI:1.6 - 1.8). In the fully adjusted model, the participants in the highest tertile had persistently greater risk of CVD (HR: 1.3, 95% CI: 1.2 - 1.4) and mortality (HR: 1.6, 95% CI: 1.5-1.7). In the continuous approach, for each 1-SD increment in BMI variability, the HRs for CVD incidence and mortality were 1.3 (95%CI: 1.2 - 1.4) and 1.7 (95%CI: 1.6 - 1.8), respectively. The subgroup analyses exhibited no difference in the association of BMI variability with CVD and mortality between the sexes and subpopulations based on obesity, diabetes, hypertension, and smoking status (**Supplementary Tables 1**, **2**).

**Table 2:** Association of BMI variability (BMI-RMSE) with all-cause mortality and CVD event.

	Events	IR (95% CI) ^*^	Model 1 HR (95% CI)	Model 2 HR (95% CI)	Model 3 HR (95% CI)
All-Cause Mortality					
BMI-RMSE (per 1 SD)	6480	15.6 (15.2-16.0)	**1.7 (1.6 - 1.8)**	**1.7 (1.6 - 1.8)**	**1.7 (1.6 - 1.8)**
BMI-RMSE Tertiles					
Tertile 1	1816	12.9 (12.4 - 13.6)	1.0 (Reference)	1.0 (Reference)	1.0 (Reference)
Tertile 2	2105	15.2 (14.6 - 15.9)	**1.2 (1.1 - 1.3)**	**1.2 (1.1 - 1.3)**	**1.2 (1.1 - 1.3)**
Tertile 3	2559	18.6 (17.9 - 19.4)	**1.7 (1.6 - 1.8)**	**1.7 (1.6 - 1.8)**	**1.6 (1.5 - 1.7)**
CVD Event					
BMI-RMSE (per 1 SD)	3900	10.2 (9.9 -10.6)	1.4 (1.3-1.5)	1.4 (1.3- 1.5)	1.3 (1.2-1.4)
BMI-RMSE Tertiles					
Tertile 1	1222	9.5 (9.0 - 10.0)	1.0 (Reference)	1.0 (Reference)	1.0 (Reference)
Tertile 2	1265	10.0 (9.4 - 10.5)	**1.1 (1.0 - 1.2)**	1.1 (1.0 - 1.2)	1.1 (1.0 - 1.2)
Tertile 3	1413	11.3 (10.7 - 11.9)	**1.4 (1.2 - 1.5)**	**1.3 (1.2 - 1.4)**	**1.3 (1.2-1.4)**

Model 1: age, sex.

Model 2: age, sex, education, smoking status, and family history of cardiovascular disease.

Model 3: age, sex, education, smoking status, family history of cardiovascular disease, baseline body mass index, fasting plasma glucose, total cholesterol, and systolic blood pressure.

BMI, body mass index; RMSE, root mean squared error; CVD, cardiovascular disease. Bolded values were statistically significant (P < .05). * IR Incidence rate per 1,000 person-years.

In the preliminary analysis, we test the mediation to see how the relationships works between the variables. So, we investigated the association between BMI variability (independent variable) and mediators (FPG, TC, and SBP), as well as the association between these mediators and CVD and mortality (dependent variable). Using the linear mixed model, the estimated beta coefficients for BMI variability as exposure and FPG, TC, and SBP as outcomes were 14.1 (95% CI: 12.8-15.4), 0.6 (95% CI: -0.6-1.9), and 3.3 (95% CI: 2.7-3.9) per 1 SD increase in BMI variability, respectively (**Table 3**). The highest BMI variability tertile was significantly associated with mean FPG and SBP with beta coefficients of 12.2 (95%CI: 11.1-13.2) and 3.1 (95%CI: 2.5-3.6), respectively. However, BMI variability was not associated with higher values of TC in the quantile approach (P value=0.2). We also performed independent risk calculations to evaluate the association of mean FPG, TC, and SBP (from first to fourth visits during the measurement period) with CVD and mortality outcomes (**Table 4**). After adjusting for potential confounders, the risk of future CVD increased for each unit increase in the mean of FPG (HR: 1.2; 95%CI: 1.1-1.3), TC (HR: 1.1; 95%CI: 1.0-1.2), and SBP (HR: 1.2; 95%CI: 1.1-1.3). The HRs for the association between the mean of FPG, TC, and SBP measurements and mortality were 1.2 (95%CI: 1.1-1.3), 1.0 (95%CI: 0.99-1.1), and 1.2 (95%CI: 1.1-1.2), respectively.

**Table 3:** Association between BMI Variability (BMI-RMSE) and time-serial measures of cardiometabolic risk factors using linear mixed model.

	Fasting plasma glucose		Total cholesterol		Systolic blood pressure	
	β (95% CI)	P value	β (95% CI)	P-value	β (95% CI)	P-value
**BMI-RMSE (per 1 SD)**	14.1 (12.8-15.4)	<0.001	0.6 (-0.6 - 1.9)	0.33	3.3 (2.7 - 3.9)	0.001
BMI-RMSE Tertiles						
Tertile 1	Reference		Reference		Reference	
Tertile 2	3.7 (2.7 - 4.8)	<0.001	1.1 (-0.03 - 2.2)	0.06	1.1 (0.5 - 1.7)	<0.001
Tertile 3	12.2 (11.1 - 13.2)	<0.001	0.7 (-0.4 - 1.9)	0.2	3.1 (2.5 - 3.6)	<0.001

Adjusted for age, sex, education, smoking status, family history of cardiovascular disease.

BMI, body mass index; RMSE, root mean squared error.

**Table 4:** Associations between the mean values of cardiovascular risk factors during the measurement period and CVD event and all-cause mortality.

	Model 1 HR (95% CI)	Model 2 HR (95% CI)	Model 3 HR (95% CI)
All-Cause Mortality			
Mean fasting plasma glucose	**1.3 (1.2 - 1.4)**	**1.3 (1.2 - 1.4)**	**1.2 (1.2 - 1.3)**
Mean systolic blood pressure	**1.2 (1.1 - 1.3)**	**1.2 (1.1 - 1.2)**	**1.2 (1.1 - 1.2)**
Mean total cholesterol	1.0 (0.99 - 1.1)	1.0 (0.99 - 1.1)	1.0 (0.99 - 1.1)
CVD Event			
Mean fasting plasma glucose	**1.3 (1.2 - 1.4)**	**1.3 (1.2 - 1.4)**	**1.2 (1.1 - 1.3)**
Mean systolic blood pressure	**1.3 (1.2 - 1.4)**	**1.3 (1.2 - 1.4)**	**1.2 (1.1 - 1.3)**
Mean total cholesterol	**1.1 (1.0 - 1.2)**	**1.1 (1.0 - 1.2)**	**1.1 (1.0 - 1.2)**

Model 1: age, sex.

Model 2: age, sex, education, smoking status, and family history of cardiovascular disease.

Model 3: age, sex, education, smoking status, family history of cardiovascular disease, baseline body mass index, fasting plasma glucose, total cholesterol, and systolic blood pressure.

CVD, cardiovascular disease. Bolded values were statistically significant (P < .05).

**Figure 2:**
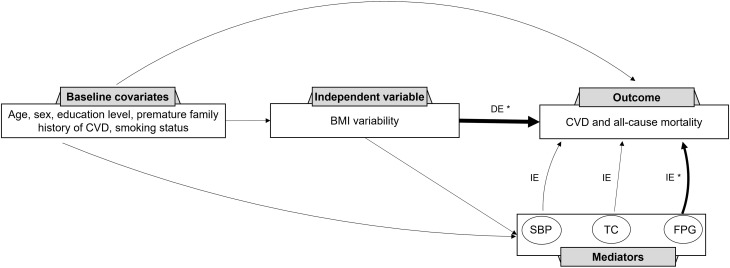
Direct Acyclic Graph (DAG) for the contribution of systolic blood pressure, total cholesterol, and fasting plasma glucose to the association between BMI variability and CVD and mortality. CVD, cardiovascular disease; BMI, body mass index; SBP, systolic blood pressure; TC, total cholesterol; FPG, fasting plasma glucose; DE, direct effect; IE, indirect effect. * Significant association to the outcome.

The relationship between BMI variability and CVD and mortality was determined considering the mediating effects of the mean values of FPG, TC, and SBP using the four-way effect decomposition (**Table 5**, **Figure 2**). After considering both the mediations and interactions, BMI variability was associated significantly with CVD (HR: 1.10, 95% CI: 1.05-1.14) and mortality (HR: 1.28, 95% CI: 1.24-1.33). For the outcome of mortality, when investigating FPG as a mediator, BMI variability was responsible for 92%, and FPG accounted for 8% of the total effect (P value<0.001), while TC and SBP were not significant mediators in the relationship (P value=0.5). The HR of the direct effect of the BMI variability on CVD was 1.08 (95% CI: 1.03-1.12), explaining 75.7% of the overall effect. The indirect effect of BMI variability via FPG as the only significant mediator was 1.02 (95%CI: 1.02-1.03) indicating 24.3% of the relationship between BMI variability and future CVD.

**Table 5:** Mediation analysis of the association of BMI variability (RMSE) with CVD event and all-cause mortality.

	BMI variability mediation with	HR (95% CI)	P-value	Mediation (%)
**All-Cause Mortality**	**Mean fasting plasma glucose**			
	Total Effect	1.28 (1.24 - 1.33)	<0.001	100%
	Direct Effect	1.26 (1.22 - 1.31)	<0.001	92%
	Indirect Effect	1.02 (1.00-1.03)	<0.001	8%
	**Mean total cholesterol**			
	Total Effect	1.27 (1.23-1.31)	<0.001	100%
	Direct Effect	1.26 (1.23-1.31)	<0.001	99.8%
	Indirect Effect	1.00 (1.00-1.00)	0.5	0.2%
	**Mean systolic blood pressure**			
	Total Effect	1.27 (1.23-1.31)	<0.001	100%
	Direct Effect	1.27 (1.23-1.31)	<0.001	99.6%
	Indirect Effect	1.00 (1.00-1.00)	0.5	0.4%
**CVD Event**	**Mean fasting plasma glucose**			
	Total Effect	1.10 (1.05-1.14)	<0.001	100%
	Direct Effect	1.08 (1.03-1.12)	<0.001	75.7%
	Indirect Effect	1.02 (1.02-1.03)	<0.001	24.3%
	**Mean total cholesterol**			
	Total Effect	1.10 (1.05-1.14)	<0.001	100%
	Direct Effect	1.09 (1.06-1.15)	<0.001	97.1%
	Indirect Effect	1.01(1.00-1.00)	0.2	2.9%
	**Mean systolic blood pressure**			
	Total Effect	1.10 (1.06-1.14)	<0.001	100%
	Direct Effect	1.10 (1.06-1.15)	<0.001	99.9%
	Indirect Effect	1.00(1.00-1.00)	0.8	0.1%

Adjusted for age, sex, education, smoking status, family history of cardiovascular disease.

CVD, cardiovascular disease.

## Discussion

This pooled analysis of cohort studies investigated the association between BMI variability and CVD and mortality over two decades. After adjusting for potential confounders, highest compared to the lowest BMI variability tertile was associated with a 30% and 60% increased risk of CVD and mortality, respectively. There was no significant difference in this association based on sex, obesity, smoking status, presence of diabetes, or hypertension. Subsequently, the mediating and interaction effects of the three important cardiometabolic factors, including FPG, SBP, and TC, were explored. We estimated that FPG mediated 24% of the excess risk for CVD and 8% of the excess risk for mortality. On the other hand, SBP and TC were not significant mediators in the association of BMI variability with CVD and mortality.

There is an ongoing controversy surrounding the impact of weight variability on CVD and mortality, especially among different subgroups. Several studies have suggested a link between weight fluctuation and a higher likelihood of future CVD and mortality (17), while others have not found any association (26, 27). Additionally, contrary findings have also emerged, suggesting that weight fluctuation could provide protection against CVD (14, 18). The underlying pathophysiology in the relationship between weight variability and CVD and mortality is not yet understood. Weight variability may be an indicator of underlying metabolic dysfunction (e.g., insulin resistance and inflammation) (28–30). It is also suggested that high variations in weight may lead to loss of muscle mass and an increase in fat mass (31, 32), increasing the risk of chronic diseases such as CVD.

Our findings suggested that high BMI variability was associated with an increased risk of future CVD and mortality, and this association was not significantly different among subpopulations (**Supplementary Tables 1**, **2**), which is consistent with the latest report by Zou et al. (17). However, it is noteworthy that high BMI fluctuation did not significantly increase the risk of future CVD in obese participants, while it increased the risk of all-cause mortality. BMI variability was associated with CVD and mortality in participants with and without diabetes.

In the current study, we determined the direct effect of BMI variability on CVD and mortality and quantified how much of the effect is mediated through FPG, SBP, and TC. Using a linear mixed model, after adjusting for confounders, we found a significant relationship between BMI variability and the time-serial values of FPG, TC, and SBP. Recent animal studies support our findings by demonstrating the adverse impact of weight cycling on glucose, insulin, and inflammatory markers’ levels (29, 33, 34). Weight variability is suggested to contribute to adipocyte enlargement and an increase in lipogenic enzymes, myristic acid, palmitic acid, palmitoleic acid, and stearic acid, resulting in glucose metabolism impairment (35, 36). Previous studies have found an independent association between high weight variability and incident diabetes (37–39), although only a few prospective studies have examined the correlation of BMI variability with the slope and alterations in cardiovascular risk factors using multiple linear regression (19, 20, 40). These studies suggested that BMI variability was associated with HbA1C levels but not SBP or TC. Notably, the impact of BMI variability on FPG was not explored. The discrepancy in the results regarding the association of BMI variability with TC and SBP may stem from differences in BMI variability calculation methods, study populations, analytical approaches, and follow-up duration. Our study, in contrast to previous ones, consisted of a large sample size.

The results of the mediation analysis revealed that 24% of the excess risk of BMI variability for CVD and 8% for mortality was mediated by FPG, while TC and SBP did not demonstrate significant mediation effects. A pooled cohort analysis conducted by the global burden of diseases reported that more than 44% of the excess risk of high baseline BMI (being overweight or obese) was mediated through FPG, TC, and SBP (41). Our study is the first to analyze the mediation effect of these risk factors in the association of BMI variability and adverse health outcomes. The non-significant mediation effect of TC and SBP in our study may be due to their weaker association with BMI variability compared to FPG (**Table 3**). These findings provide new insight into understanding the complex relationship between BMI variability, major cardiovascular risk factors, and CVD and mortality.

In the current study, for the first time, the direct and indirect effect of BMI variability on CVD and mortality was investigated in a pooled cohort analysis of three large longitudinal population-based studies with long follow-ups. Many studies resort to simple statistical methods to measure BMI variability, overlooking the non-linear nature of BMI trend and mistakenly perceiving it as fluctuation (24). To address this, we employed the RMSE within a random coefficient model to differentiate between true non-linear changes in BMI and BMI fluctuation. There are also some limitations in this study. Our study included the BMI measurements taken in the clinic and the BMI measurements were not self-reported although it is noteworthy that the measurements in the clinic are done with unified standard protocols. We did not assess whether the participants had intentions of losing or gaining weight. However, the primary goal was that unintentional weight loss may have been an indicator of underlying diseases. Also, our findings solely pertained to BMI variability and lacked information regarding alterations in fat mass, muscle mass, and change in body composition. Future research should prioritize lifelong maintenance of body weight and reduction of sporadic weight fluctuations, especially in the context of primary cardiac prevention. This approach can help in sustaining healthy weight levels and minimizing associated health risks.

In conclusion, BMI variability is an independent predictor for incident CVD and mortality and there is no significant difference in this association across subpopulations. FPG mediates 24% and 8% of excess risk of BMI variability for the development of CVD and mortality, respectively; however, SBP and TC are not significant mediators in this association. These findings present new potential implications for preventing CVD and mortality in individuals with high BMI variability. Modern cardiometabolic interventions focusing on glycemic monitoring and management might help reduce the risk of future CVD and mortality during frequent weight loss attempts.

## Data availability statement

The data analyzed in this study is subject to the following licenses/restrictions: Datasets generated during and/or analyzed during the current study are not publicly available due to institutional policies but are available from the corresponding author on reasonable request. Requests to access these datasets should be directed to amouzegar@endocrine.ac.ir.

## Ethics statement

The studies involving humans were approved by the National Research Council of the Islamic Republic of Iran (IR.SBMU.ENDOCRINE.REC.1402.060), the Human Research Review Committee of the Endocrine Research Center, Shahid Beheshti University of Medical Sciences, Tehran, Iran. The studies were conducted in accordance with the local legislation and institutional requirements. The participants provided their written informed consent to participate in this study.

## Author contributions

LM: Conceptualization, Methodology, Writing – original draft, Writing – review & editing. MH: Conceptualization, Data curation, Formal Analysis, Investigation, Methodology, Visualization, Writing – original draft, Writing – review & editing. SM: Data curation, Formal Analysis, Methodology, Visualization, Writing – original draft. DK: Methodology, Writing – review & editing. FA: Supervision, Writing – review & editing. MB: Supervision, Writing – review & editing. AA: Project administration, Writing – review & editing.

## References

[1] Mongraw-Chaffin ML Peters SAE Huxley RR Woodward M. The sex-specific association between BMI and coronary heart disease: a systematic review and meta-analysis of 95 cohorts with 1·2 million participants. Lancet Diabetes Endocrinol. (2015) 3:437–49. doi: 10.1016/S2213-8587(15)00086-8 PMC4470268 25960160

[2] Di Angelantonio E Bhupathiraju SN Wormser D Gao P Kaptoge S de Gonzalez AB. Body-mass index and all-cause mortality: individual-participant-data meta-analysis of 239 prospective studies in four continents. Lancet. (2016) 388:776–86. doi: 10.1016/S0140-6736(16)30175-1 PMC4995441 27423262

[3] Jensen MD Ryan DH Apovian CM Ard JD Comuzzie AG Donato KA. 2013 AHA/ACC/TOS guideline for the management of overweight and obesity in adults. Circulation. (2014) 129:S102–38. doi: 10.1161/01.cir.0000437739.71477.ee PMC5819889 24222017

[4] Wing RR Phelan S. Long-term weight loss maintenance. Am J Clin Nutr. (2005) 82:222s–5s. doi: 10.1093/ajcn.82.1.222S 16002825

[5] Santos I Sniehotta FF Marques MM Carraça EV Teixeira PJ. Prevalence of personal weight control attempts in adults: a systematic review and meta-analysis. Obes Rev. (2017) 18:32–50. doi: 10.1111/obr.12466 27653242 PMC5215364

[6] Ochner CN Barrios DM Lee CD Pi-Sunyer FX. Biological mechanisms that promote weight regain following weight loss in obese humans. Physiol Behav. (2013) 120:106–13. doi: 10.1016/j.physbeh.2013.07.009 PMC3797148 23911805

[7] Lavie CJ Laddu D Arena R Ortega FB Alpert MA Kushner RF. Reprint of: healthy weight and obesity prevention: JACC health promotion series. J Am Coll Cardiol. (2018) 72:3027–52. doi: 10.1016/j.jacc.2018.10.024 30522635

[8] Elagizi A Kachur S Lavie CJ Carbone S Pandey A Ortega FB. An overview and update on obesity and the obesity paradox in cardiovascular diseases. Prog Cardiovasc Dis. (2018) 61:142–50. doi: 10.1016/j.pcad.2018.07.003 29981771

[9] Hartmann-Boyce J Theodoulou A Oke JL Butler AR Bastounis A Dunnigan A. Long-term effect of weight regain following behavioral weight management programs on cardiometabolic disease incidence and risk: systematic review and meta-analysis. Circulation: Cardiovasc Qual Outcomes. (2023) 16:e009348. doi: 10.1161/CIRCOUTCOMES.122.009348 PMC10106109 36974678

[10] Huang S Shi K Ren Y Wang J Yan WF Qian WL. Association of magnitude of weight loss and weight variability with mortality and major cardiovascular events among individuals with type 2 diabetes mellitus: a systematic review and meta-analysis. Cardiovasc Diabetol. (2022) 21:78. doi: 10.1186/s12933-022-01503-x 35578337 PMC9112517

[11] Powell-Wiley TM Poirier P Burke LE Després J-P Gordon-Larsen P Lavie CJ. Obesity and cardiovascular disease: A scientific statement from the american heart association. Circulation. (2021) 143:e984–e1010. doi: 10.1161/CIR.0000000000000973 33882682 PMC8493650

[12] Massey RJ Siddiqui MK Pearson ER Dawed AY. Weight variability and cardiovascular outcomes: a systematic review and meta-analysis. Cardiovasc Diabetol. (2023) 22:5. doi: 10.1186/s12933-022-01735-x 36624453 PMC9830835

[13] Kaze AD Santhanam P Erqou S Ahima RS Bertoni AG Echouffo-Tcheugui JB. Body weight variability and risk of cardiovascular outcomes and death in the context of weight loss intervention among patients with type 2 diabetes. JAMA Network Open. (2022) 5:e220055–e220055. doi: 10.1001/jamanetworkopen.2022.0055 35179583 PMC8857684

[14] Jeong S Choi S Chang J Kim K Kim SM Hwang SY. Association of weight fluctuation with cardiovascular disease risk among initially obese adults. Sci Rep. (2021) 11:10152. doi: 10.1038/s41598-021-89666-7 33980955 PMC8115677

[15] Oh TJ Moon JH Choi SH Lim S Park KS Cho NH. Body-weight fluctuation and incident diabetes mellitus, cardiovascular disease, and mortality: A 16-year prospective cohort study. J Clin Endocrinol Metab. (2019) 104:639–46. doi: 10.1210/jc.2018-01239 30500906

[16] Sponholtz TR van den Heuvel ER Xanthakis V Vasan RS. Association of variability in body mass index and metabolic health with cardiometabolic disease risk. J Am Heart Assoc. (2019) 8:e010793. doi: 10.1161/JAHA.118.010793 31025893 PMC6509716

[17] Zou H Yin P Liu L Liu W Zhang Z Yang Y. Body-weight fluctuation was associated with increased risk for cardiovascular disease, all-cause and cardiovascular mortality: A systematic review and meta-analysis. Front Endocrinol. (2019) 10. doi: 10.3389/fendo.2019.00728 PMC6856014 31787929

[18] Mehran L Honarvar M Masoumi S Khalili D Amouzegar A Azizi F. Weight fluctuation, mortality, and cardiovascular disease in adults in 18 years of follow-up: Tehran Lipid and Glucose Study. J Endocrinol Invest. (2023) 46:37–49. doi: 10.1007/s40618-022-01881-9 35921037

[19] Nakanishi N Nakamura K Suzuki K Tatara K. Effects of weight variability on cardiovascular risk factors; a study of nonsmoking Japanese male office workers. Int J Obes. (2000) 24:1226–30. doi: 10.1038/sj.ijo.0801389 11033995

[20] Taylor CB Jatulis DE Fortmann SP Kraemer HC. Weight variability effects: A prospective analysis from the stanford five-city project. Am J Epidemiol. (1995) 141:461–5. doi: 10.1093/oxfordjournals.aje.a117448 7879790

[21] Investigators A. The atherosclerosis risk in communit (ARIC) study: design and objectives. Am J Epidemiol. (1989) 129:687–702. doi: 10.1093/oxfordjournals.aje.a115184 2646917

[22] Bild DE Bluemke DA Burke GL Detrano R Diez Roux AV Folsom AR. Multi-ethnic study of atherosclerosis: objectives and design. Am J Epidemiol. (2002) 156:871–81. doi: 10.1093/aje/kwf113 12397006

[23] Azizi F Ghanbarian A Momenan AA Hadaegh F Mirmiran P Hedayati M. Prevention of non-communicable disease in a population in nutrition transition: Tehran Lipid and Glucose Study phase II. Trials. (2009) 10:5. doi: 10.1186/1745-6215-10-5 19166627 PMC2656492

[24] Cologne J Takahashi I French B Nanri A Misumi M Sadakane A. Association of weight fluctuation with mortality in Japanese adults. JAMA Network Open. (2019) 2:e190731–e190731. doi: 10.1001/jamanetworkopen.2019.0731 30874785 PMC6484619

[25] Discacciati A Bellavia A Lee JJ Mazumdar M Valeri L. Med4way: a Stata command to investigate mediating and interactive mechanisms using the four-way effect decomposition. Int J Epidemiol. (2018) 48(1):15–20. doi: 10.1093/ije/dyy236 30452641

[26] Lissner L Andres R Muller DC Shimokata H. Body weight variability in men: metabolic rate, health and longevity. Int J Obes. (1990) 14:373–83. 2361814

[27] Field AE Malspeis S Willett WC. Weight cycling and mortality among middle-aged or older women. Arch Intern Med. (2009) 169:881–6. doi: 10.1001/archinternmed.2009.67 PMC2764352 19433700

[28] Yatsuya H Tamakoshi K Yoshida T Hori Y Zhang H Ishikawa M. Association between weight fluctuation and fasting insulin concentration in Japanese men. Int J Obes Relat Metab Disord. (2003) 27:478–83. doi: 10.1038/sj.ijo.0802221 12664081

[29] Li X Jiang L Yang M Wu YW Sun JZ. Impact of weight cycling on CTRP3 expression, adipose tissue inflammation and insulin sensitivity in C57BL/6J mice. Exp Ther Med. (2018) 16:2052–9. doi: 10.3892/etm PMC6122336 30186439

[30] Tamakoshi K Yatsuya H Kondo T Ishikawa M Zhang H Murata C. Long-term body weight variability is associated with elevated C-reactive protein independent of current body mass index among Japanese men. Int J Obes Relat Metab Disord. (2003) 27:1059–65. doi: 10.1038/sj.ijo.0802386 12917711

[31] Chaston TB Dixon JB. Factors associated with percent change in visceral versus subcutaneous abdominal fat during weight loss: findings from a systematic review. Int J Obes (Lond). (2008) 32:619–28. doi: 10.1038/sj.ijo.0803761 18180786

[32] MacLean PS Higgins JA Giles ED Sherk VD Jackman MR. The role for adipose tissue in weight regain after weight loss. Obes Rev. (2015) 16 Suppl 1:45–54. doi: 10.1111/obr.12255 25614203 PMC4371661

[33] Schofield SE Parkinson JR Henley AB Sahuri-Arisoylu M Sanchez-Canon GJ Bell JD. Metabolic dysfunction following weight cycling in male mice. Int J Obes (Lond). (2017) 41:402–11. doi: 10.1038/ijo.2016.193 PMC5344184 27840414

[34] Simonds SE Pryor JT Cowley MA. Repeated weight cycling in obese mice causes increased appetite and glucose intolerance. Physiol Behav. (2018) 194:184–90. doi: 10.1016/j.physbeh.2018.05.026 29842854

[35] Sea MM Fong WP Huang Y Chen ZY. Weight cycling-induced alteration in fatty acid metabolism. Am J Physiol Regul Integr Comp Physiol. (2000) 279:R1145–55. doi: 10.1152/ajpregu.2000.279.3.R1145 10956277

[36] Eguchi K Manabe I Oishi-Tanaka Y Ohsugi M Kono N Ogata F. Saturated fatty acid and TLR signaling link β cell dysfunction and islet inflammation. Cell Metab. (2012) 15:518–33. doi: 10.1016/j.cmet.2012.01.023 22465073

[37] Prattichizzo F Frigé C La Grotta R Ceriello A. Weight variability and diabetes complications. Diabetes Res Clin Pract. (2023) 199:110646. doi: 10.1016/j.diabres.2023.110646 37001818

[38] Hiroshi O Masahide H Momoko H Kazushiro K Hiroaki M Masato I. Association between variability in body mass index and development of type 2 diabetes: Panasonic cohort study. BMJ Open Diabetes Res &amp; Care. (2021) 9:e002123. doi: 10.1136/bmjdrc-2021-002123 PMC8070854 33888538

[39] Mehran L Mousapour P Khalili D Cheraghi L Honarvar M Amouzegar A. BMI variability and incident diabetes mellitus, Tehran Lipid and Glucose Study (TLGS). Sci Rep. (2022) 12:18370. doi: 10.1038/s41598-022-22817-6 36319811 PMC9626493

[40] Turicchi J O’Driscoll R Horgan G Duarte C Santos I Encantado J. Body weight variability is not associated with changes in risk factors for cardiometabolic disease. Int J Cardiol Hypertens. (2020) 6:100045. doi: 10.1016/j.ijchy.2020.100045 33447771 PMC7803052

[41] Global Burden of Metabolic Risk Factors for Chronic Diseases Collaboration (BMI Mediated Effects), Lu Y, Hajifathalian K, Ezzati M, Woodward M, Rimm EB, et al. Metabolic mediators of the effects of body-mass index, overweight, and obesity on coronary heart disease and stroke: a pooled analysis of 97 prospective cohorts with 1·8 million participants. Lancet. (2014) 383:970–83. doi: 10.1016/S0140-6736(13)61836-X PMC3959199 24269108

