# Supplementary material for: The association of body mass index variability with cardiovascular disease and mortality: a mediation analysis of pooled cohorts
Source: Front Endocrinol (Lausanne). 2024 May 13;15:1345781. doi: 10.3389/fendo.2024.1345781 (PMC11128653; doi:10.3389/fendo.2024.1345781)
Supplement: Supplementary file 1 [file Table_1.docx]

Supplementary Table 1. Association between BMI variability (BMI-RMSE) and all-cause mortality

|  | **BMI variability tertiles** | | | **P Value Interaction** |
| --- | --- | --- | --- | --- |
|  | **Tertile 1** | **Tertile 2** | **Tertile 3** |  |
| Sex |  |  |  | 0.35 |
| Male | 1.0 (Reference) | 1.19(1.10-1.30) | 1.65(1.51-1.81) |  |
| Female | 1.0 (Reference) | 1.26(1.14-1.39) | 1.54(1.39-1.69) |  |
| BMI |  |  |  | 0.18 |
| < 30 | 1.0 (Reference) | 1.25 (1.16-1.34) | 1.58 (1.47-1.71) |  |
| ≥ 30 | 1.0 (Reference) | 1.02 (0.89-1.19) | 1.36 (1.20-1.56) |  |
| Smoking |  |  |  | 0.45 |
| Never | 1.0 (Reference) | 1.21 (1.13-1.30) | 1.57 (1.46-1.68) |  |
| Current | 1.0 (Reference) | 1.19 (0.98-1.45) | 1.52 (1.23-1.87) |  |
| Diabetes |  |  |  | 0.36 |
| Yes | 1.0 (Reference) | 1.21 (1.01-1.46) | 1.51 (1.26-1.81) |  |
| No | 1.0 (Reference) | 1.19 (1.11-1.28) | 1.54 (1.44-1.65) |  |
| Hypertension |  |  |  | 0.70 |
| Yes | 1.0 (Reference) | 1.15 (1.04-1.27) | 1.43 (1.29-1.59) |  |
| No | 1.0 (Reference) | 1.22(1.13-1.33) | 1.64 (1.51-1.78) |  |

Adjusted for age, sex, education, smoking status, family history of cardiovascular disease, baseline body mass index, fasting plasma sugar, total cholesterol, and systolic blood pressure
Abbreviations: RMSE, root mean squared error

Supplementary Table 2. Association between BMI variability (BMI-RMSE) and CVD event

|  | **BMI variability tertiles** | | | P Value Interaction |
| --- | --- | --- | --- | --- |
|  | **Tertile 1** | **Tertile 2** | **Tertile 3** |  |
| Sex |  |  |  | 0.9 |
| Male | 1.0 (Reference) | 1.08 (0.97-1.19) | 1.24 (1.11-1.39) |  |
| Female | 1.0 (Reference) | 1.13 (0.99-1.29) | 1.33 (1.17-1.52) |  |
| BMI |  |  |  | 0.7 |
| < 30 | 1.0 (Reference) | 1.08(0.99-1.18) | 1.17(1.06-1.29) |  |
| ≥ 30 | 1.0 (Reference) | 0.94 (0.79-1.12) | 1.10 (0.94-1.29) |  |
| Smoking |  |  |  | 0.2 |
| Never | 1.0 (Reference) | 1.01(0.92-1.10) | 1.12(1.02-1.22) |  |
| Current | 1.0 (Reference) | 1.36(1.09-1.70) | 1.42(1.12-1.79) |  |
| Diabetes |  |  |  | 0.7 |
| Yes | 1.0 (Reference) | 1.01(0.88-1.13) | 1.19(1.05-1.35) |  |
| No | 1.0 (Reference) | 1.09(0.98-1.20) | 1.19(1.07-1.32) |  |
| Hypertension |  |  |  | 0.3 |
| Yes | 1.0 (Reference) | 1.11(0.96-1.27) | 1.13(0.97-1.31) |  |
| No | 1.0 (Reference) | 1.03(0.93-1.13) | 1.17(1.06-1.29) |  |

Adjusted for age, sex, education, smoking status, family history of cardiovascular disease, baseline body mass index, fasting plasma sugar, total cholesterol, and systolic blood pressure
Abbreviations: RMSE, root mean squared error
